# SARS-CoV-2 Viroporin E Induces Ca^2+^ Release and Neuron Cell Death in Primary Cultures of Rat Hippocampal Cells Aged In Vitro

**DOI:** 10.3390/ijms25126304

**Published:** 2024-06-07

**Authors:** Sara López-Vázquez, Carlos Villalobos, Lucía Núñez

**Affiliations:** 1 Excellence Unit, Institute of Biomedicine and Molecular Genetics of Valladolid (IBGM), University of Valladolid and Spanish National Research Council (CSIC), 47003 Valladolid, Spain; sara.lopez.vazquez@uva.es (S.L.-V.); nunezl@uva.es (L.N.); 2 Department of Biochemistry and Molecular Biology and Physiology, School of Medicine, University of Valladolid, 47005 Valladolid, Spain

**Keywords:** SARS-CoV-2, COVID-19, viroporins, hippocampal neurons, aging, calcium

## Abstract

The COVID-19 pandemic was caused by infection with Severe Acute Respiratory Syndrome Coronavirus 2 (SARS-CoV-2), which may lead to serious respiratory, vascular and neurological dysfunctions. The SARS-CoV-2 envelope protein (E protein) is a structural viroporin able to form ion channels in cell membranes, which is critical for viral replication. However, its effects in primary neurons have not been addressed. Here we used fluorescence microscopy and calcium imaging to study SARS-CoV-2 viroporin E localization and the effects on neuron damage and intracellular Ca^2+^ homeostasis in a model of rat hippocampal neurons aged in vitro. We found that the E protein quickly enters hippocampal neurons and colocalizes with the endoplasmic reticulum (ER) in both short-term (6–8 days in vitro, DIV) and long-term (20–22 DIV) cultures resembling young and aged neurons, respectively. Strikingly, E protein treatment induces apoptosis in aged neurons but not in young neurons. The E protein induces variable increases in cytosolic Ca^2+^ concentration in hippocampal neurons. Ca^2+^ responses to the E protein are due to Ca^2+^ release from intracellular stores at the ER. Moreover, E protein-induced Ca^2+^ release is very small in young neurons and increases dramatically in aged neurons, consistent with the enhanced Ca^2+^ store content in aged neurons. We conclude that the SARS-CoV-2 E protein quickly translocates to ER endomembranes of rat hippocampal neurons where it releases Ca^2+^, probably acting like a viroporin, thus producing Ca^2+^ store depletion and neuron apoptosis in aged neurons and likely contributing to neurological damage in COVID-19 patients.

## 1. Introduction

COVID-19 is an infectious disease caused by the novel Severe Acute Respiratory Syndrome Coronavirus 2 (SARS-CoV-2), which emerged in late 2019 and led to the declaration of a pandemic in 2020. COVID-19 symptoms are greatly variable depending on the patient. Usually, advanced age or underlying health conditions imply a poor prognosis for the illness. Given the large magnitude of this disease, symptoms were compiled, and it was found that there are widespread neurological complications associated with SARS-CoV-2 infection. Among them are headaches, anosmia, dizziness, seizures, impaired consciousness, encephalitis, and acute cerebrovascular diseases, including stroke, venous sinus thrombosis, and intracerebral hemorrhage [1,2,3]. The long-term functional outcome was favorable in most neuro-COVID disorders, although mild symptoms commonly lasted more than 6 months after infection. These facts highlight the need to study the COVID-19 disease from the nervous system perspective to elucidate the mechanisms by which the SARS-CoV-2 virus may negatively affect neuronal function.

The molecular structure of SARS-CoV-2, like other coronaviruses, is characterized by being spherical, measuring 120–160 nm across [4]. At the structural level, SARS-CoV-2 acquires a lipid bilayer envelope from the host cell it infects. The envelope assembles four structural proteins [5]. They include the spike protein, or S protein, which binds with high affinity to the human angiotensin-converting enzyme 2 (ACE2), initiating endocytosis and facilitating virus infection [6]. ACE2 is expressed in various organs, including the brain [7]. The second protein is the membrane protein, or M protein, which plays an essential role in viral morphogenesis [8]. The third protein is the envelope protein, or E protein. The most widely recognized function of the E protein is to participate in the encapsulation and budding at the membrane of the endoplasmic reticulum (ER) Golgi intermediate compartment (ERGIC) [9,10]. Nonetheless, the E protein has additional roles under scrutiny. Finally, the fourth protein is the nucleocapsid protein, or N protein, which plays crucial roles in the virus life cycle, such as viral RNA replication and transcription [11].

Highly infectious viruses such as the human immunodeficiency virus (HIV), Ebola virus, or hepatitis C virus encode proteins that function as ion channels. Therefore, the disruption of intracellular ion homeostasis in the infected cell may be an important consequence during viral infection. Viroporins are small hydrophobic proteins that oligomerize to form hydrophilic pores in the membrane of host cells. Their ion channel activity increases cell membrane permeability, modifies Ca^2+^ homeostasis, induces membrane remodeling, and disrupts glycoprotein trafficking [12]. Further, the contribution of viroporins to the viral life cycle involves virion assembly and release from infected cells [13,14]. The classification of viroporins consists of three classes, I to III, named according to the number of transmembrane domains [15]. Class III viroporins have three transmembrane domains flanked by a short N-terminal domain that faces the ER lumen and a longer C-terminal domain located in the cytosol.

The SARS-CoV-2 virus genome encodes three viroporins. The first of these is the 3a protein. This class III viroporin with a length of 274 amino acids may form Ca^2+^-permeable non-selective cation channels. The 3a protein plays an important role in replication and virulence [16]. The second one is the 8a protein. This 39-amino-acid-long protein contains a single transmembrane domain and may form weak cation-selective channels found within mitochondria [17]. Its functions include playing a minor role in viral replication and inducing apoptosis through a mitochondrion-dependent pathway [18]. Finally, the third one is the E protein, an integral membrane protein of 75 amino acids categorized as a class I viroporin. E protein channel activity has been associated with the flux of cations such as Na^+^, K^+^, and even Ca^2+^. It is present in low quantities in viral particles but highly expressed in infected cells, where it is mainly located in the Golgi, ER, and ERGIC [19,20]. The size of CoV E proteins ranges from 8.4 to 12 kDa that may form dimers, trimers or pentamers. SARS-CoV and SARS-CoV-2 are composed of 76 and 75 amino acids, respectively. Both of them consist of three main domains: one TMD (residues 17–37), an intermediate helical domain, and N- and C-terminal domains. The N-terminus of the CoV E protein has a negatively charged hydrophilic amino terminal. In addition, the protein has an uncharged (nonpolar) hydrophobic TMD. The C-terminus shows low hydrophobicity, but it also has 20 hydrophobic residues out of a total of 37 amino acids that are positively charged. The E protein of SARS-CoV-2 differs in only three amino acid substitutions and one deletion. The E protein may not exhibit a uniform membrane topology, and its orientation depends on the level of protein expression or oligomerization [20].

The E protein has been involved in multiple roles throughout viral infection. In the first place, it may be involved in morphogenesis and viral assembly. The E protein causes bending of the surrounding lipid bilayer [21] and regulates the fluidity of the viral envelope. Thus, the E protein is involved in the formation of virus-like particles (VLPs) and viral budding [22,23]. In second place is virulence. The ion channel activity of the E protein plays a critical role in virulence since it is related to edema accumulation and inflammasome activation through IL-1β, accompanied by TNF and IL-6, cytokines that promote the progression of lung damage and ARDS, ultimately leading to death [19]. Finally, the E protein has been involved in neurotropism. Interestingly, the SARS-CoV-2 virus has been described as a pathogen with neurotropism, in other words, with affinity for nervous tissue [24]. In this regard, two mechanisms of nervous infection have been described. Firstly, SARS-CoV-2 infection triggers inflammation and a massive release of cytokines, chemokines, and other inflammatory signals which can compromise the integrity of the blood–brain barrier, allowing the virus to enter the central nervous system (CNS) via bloodstream circulation [7]. Secondly, axonal and transneuronal transport spread from olfactory and trigeminal nerve endings in the nasal epithelium [25].

Intracellular Ca^2+^ homeostasis plays a pivotal role in all cells and particularly neurons. For instance, rises in intracellular Ca^2+^ are involved in neurotransmitter release and synaptic plasticity. However, if Ca^2+^ responses are large and sustained enough, they may lead to neuron apoptosis. We have previously established a primary culture model of cultured hippocampal neurons to test the effects of aging on intracellular Ca^2+^ homeostasis and the influence of different neurotoxins related to neurodegenerative diseases on both intracellular Ca^2+^ homeostasis and susceptibility to neuron cell death [26,27]. Even though the possible role of the SARS-CoV-2 E protein as a viroporin has been proposed, its implication in intracellular Ca^2+^ homeostasis in primary neurons has not been established. Accordingly, we have tested the effects of the SARS-CoV-2 E protein on intracellular Ca^2+^ homeostasis in primary neurons here. In addition, given that long-term cultured neurons acquire an aging phenotype and enhanced susceptibility to damage induced by established neurotoxins [28,29], we also aimed to explore the possible contribution of aging to the effects of the E protein on intracellular Ca^2+^ and apoptosis.

## 2. Results

### 2.1. SARS-CoV-2 E Protein Is Intracellularly Located and Colocalizes with the ER

**Figure 1:**
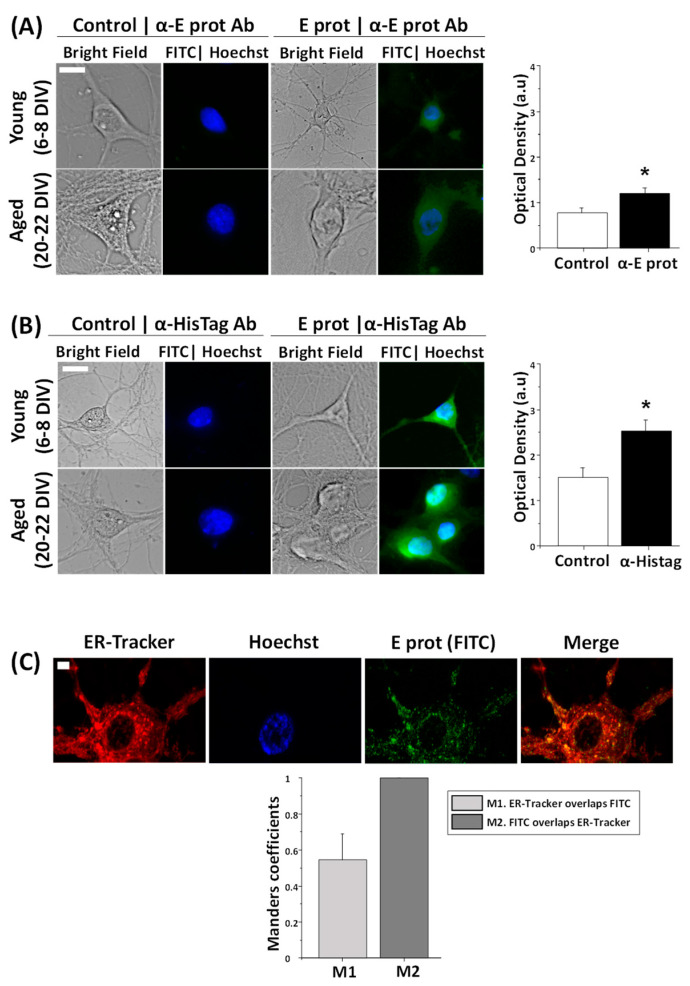
The SARS-CoV-2 E protein is intracellularly located and colocalizes with the endoplasmic reticulum in hippocampal neurons. E protein location was tested in rat hippocampal cultures treated with the vehicle (Control) or the E protein (E protein, 0.6 µg/mL) for 24 h. Immunochemistry experiments were independently performed with two different antibodies, against the E protein and HisTag. Incubation with ER-Tracker allowed one to observe endoplasmic reticulum. (**A**) Representative bright field and immunofluorescence images of neurons treated with the E protein and nuclei (FITC|Hoechst) taken from young (6–8 DIV) and aged (20–22 DIV) hippocampal neurons using antibodies against the E protein (α-E protein Ab). Alongside is the quantitative age-independent analysis of immunofluorescence intensity levels (Optical density in arbitrary units) for control and E protein (E prot), using E protein antibody. The bars represent the mean ± SEM from 133 and 165 cells from five independent experiments. The scale bar is 10 µm. * *p* < 0.05 vs. control. (**B**) The same procedure using HisTag antibody (α-HisTag Ab). Data were taken from 190 and 207 cells from five independent experiments. The scale bar is 10 µm. * *p* < 0.05 vs. control. (**C**) Representative confocal fluorescence images from endoplasmic reticulum (ER-Tracker), nuclei (Hoechst), the E protein using E protein antibody (E prot FITC) and the merge of a 21 DIV neuron. The bars represent the Manders coefficient M1 (ER-Tracker overlapping FITC) and M2 (FITC overlapping ER-Tracker) mean ± SEM from 16 cells in three independent experiments. The scale bar is 5 µm.

Protein localization is essential to clarifying the role of the E protein in cellular physiology. Therefore, the first step was to determine whether the protein acts from outside or inside the cell. For this purpose, hippocampal neurons were cultured between 6–8 DIV and 20–22 DIV and treated for 24 h in the presence of the E protein (0.6 µg/mL), or in its absence as control. Immunohistochemistry assays were performed to identify the localization of the E protein. To this end, we used two different antibodies: one against the E protein and the other against the HisTag label used to identify this protein. Cell nuclei were stained using Hoechst. Optical density measures (a.u) taken from fluorescence images show an increase in fluorescence signal in neurons treated with the E protein, regardless of whether we use E protein antibody (Figure 1A) or HisTag antibody (Figure 1B). These results suggest an intracellular location for the E protein with both antibodies when added extracellularly. Time of culture did not have an effect on fluorescence results.

According to literature, during SARS-CoV-2 infection, the E protein is located in the ER [20]. However, conventional fluorescence microscopy does not allow one to clarify the subcellular location of the E protein. Accordingly, we used confocal microscopy and ER staining together with immunofluorescence to investigate whether the E protein colocalizes with the ER in neurons exposed to free E protein as well. Hippocampal cultures were treated for 24 h with the E protein (0.6 µg/mL) and incubated with ER-Tracker and anti-E protein antibody, to target the ER in red and the E protein in green, respectively. Cell nuclei were stained using Hoechst. Fluorescence images and Manders coefficient analysis indicate a clear colocalization between the E protein and ER (Figure 1C). On one hand, M2 indicates that in every green signal there is always a red signal. As expected, wherever there is the E protein there is ER. On the other hand, M1 indicates that in not every red signal is there a green signal. This result is also expected and indicates that the E protein is not present on the entire surface of the ER but, perhaps, in specialized areas of the ER, such as the Golgi and the ERGIC.

These results indicate that extracellularly applied E protein from SARS-CoV-2 may enter neurons and locate at the ER, likely influencing the ER activities of primary neurons. These results are similar to those previously reported for other non-neuronal, SARS-CoV virus-infected cells and cells transfected with a plasmid encoding the E protein from SARS-CoV, the early variant of SARS-CoV-2 [30]. Therefore, extracellularly applied E protein may enter neurons and, perhaps, behave like viroporins produced during neuron cell infection. 

### 2.2. SARS-CoV-2 E Protein Is Toxic to Aged Rat Hippocampal Neurons but Not to Young Neurons

**Figure 2:**
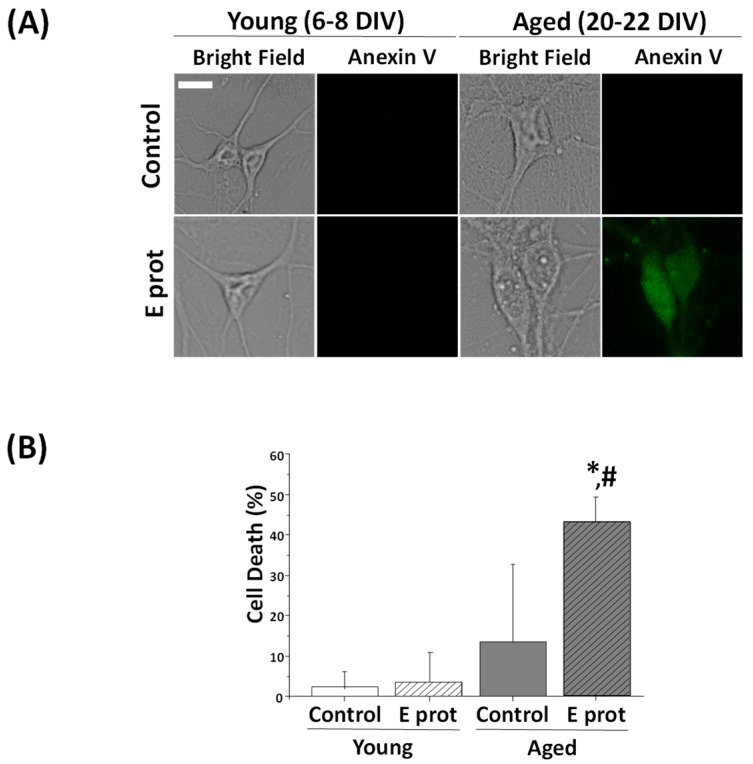
The SARS-CoV-2 E protein induces cell death in aged cultures of rat hippocampal neurons but not in young cultures. Primary hippocampal neuron cultures for 6–8 DIV (young) and 20–22 DIV (aged) were treated for 24 h in the absence or presence of the SARS-CoV-2 E protein. Apoptosis was assessed 24 h later by means of staining with Annexin V. (**A**) Representative bright field and Annexin V inmunofluorescence images of young (upper) and aged (bottom) hippocampal cultures in the presence (E prot) or absence (Control) of the E protein (0.6 μg/mL). A bar represents 10 μm and applies to all photographs. (**B**) Percentage of neuron dead cells in control and E protein-treated cells in young and aged cultures. Values represent the mean ± SEM from 865, 880, 561 and 639 cells from seven independent experiments. * *p* < 0.05 vs. control; # *p* < 0.05 vs. young cultures.

The E protein is proposed as the main element of virulence in the SARS-CoV-2 virus. Consequently, cell survival was tested in hippocampal cultures to analyze neuronal damage. Apoptosis triggers translocation of the membrane phospholipid phosphatidylserine from the inner to the outer leaflet of the membrane. Hence, phosphatidylserine expression at the external membrane was investigated by the Annexin V assay. Hippocampal neurons were cultured for 6–8 DIV or 20–22 DIV and incubated for 24 h in the presence of the E protein (0.6 µg/mL), or in its absence as control. Then, cells were subjected to fluorescence imaging and the fraction of cellular apoptosis was measured. Neurons were selected according to morphologic characteristics. Figure 2 shows that 24 h incubation with the E protein (0.6 µg/mL) increases apoptosis in aged hippocampal cultures; however, there is no significant effect on young cultures.

These results are similar to those obtained with other age-related neurotoxins, for instance, NMDA-induced neurotoxicity, lipopolysaccharide, a Toll-like receptor activator mimicking neuroinflammation, or amyloid-β peptide oligomers involved in Alzheimer’s disease. In all these cases, neurotoxins promote neuron cell death in aged neurons but not in young neurons [31,32,33], just like we have observed here with SARS-CoV-2 viroporin E.

### 2.3. SARS-CoV-2 E Protein Increases Cytosolic Ca^2+^ in Young and Aged Hippocampal Neurons

The E protein is a viroporin, which means that it can act as an ion channel. The literature associates E protein channel activity with Ca^2+^ currents. Given this fact, we wanted to know whether intracellular Ca^2+^ homeostasis in neurons could be affected by the SARS-CoV-2 Envelope protein. In addition, we proposed that the age of culture may be an important factor for the experiment, as previously reported for other neurotoxins. Hippocampal cultures, incubated for 6–8 DIV or 20–22 DIV, were loaded with fura2/AM and subjected to calcium imaging in ECM. While recording, cells were acutely exposed to the E protein (2 μg/mL), or the vehicle as control. 

**Figure 3:**
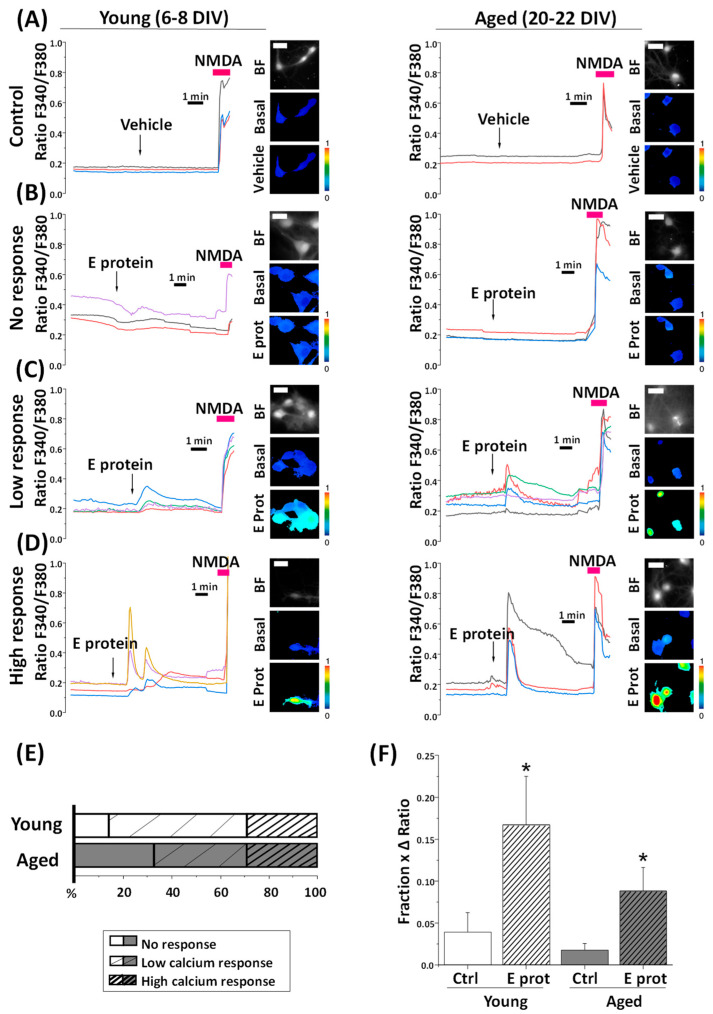
The SARS-CoV-2 E protein induces diverse cytosolic calcium responses in young and aged hippocampal neurons. Hippocampal neurons were loaded with fura2/AM and subjected to fluorescence imaging to monitoring changes in cytosolic calcium levels in response to E protein (2 μg/mL) acute treatment. Traces are representative recordings of fluorescence ratios in individual neurons for each type of response, (**A**) vehicle treatment (Control), (**B**) E protein treatment with lack of response (No response), (**C**) low calcium response (Low response), and (**D**) high calcium response (High response) in young (6–8 DIV) and aged (20–22 DIV) cultures. Neurons were identified by their morphology and N-Methyl-D-Aspartate response (NMDA, 100 µM). The pictures show bright field images (BF) and pseudocolor images of fluorescence ratios taken before (Basal) and after vehicle (Vehicle) or E protein (E prot) acute treatment in young (6–8 DIV) and aged (20–22 DIV) cultured neurons. The pseudocolor scale is shown at right. A bar represents 10 μm and applies to all photographs. (**E**) The bars represent the percentage of each type of response observed, according to the age of culture. (**F**) The bar plot represents the mean ± SEM of the “Fraction × ΔRatio” parameter, which is the product of the proportion of responsive cells and the maximum height ratio F340/F380 (Δ ratio) n = 33, 36, 58 and 96 cells from 4–12 independent experiments * *p* < 0.05 vs. control.

We observed that the addition of E protein induced a variable rise in intracellular free Ca^2+^ concentration ([Ca^2+^]_cyt_) in identified neurons. Ca^2+^ responses to the E protein are diverse among neuron cultures in both young and aged ones (Figure 3). Whereas the vehicle does not produce significant changes in [Ca^2+^]_cyt_ levels, the E protein can induce either a slight increase (low response), a large increase (high response), or simply an absence of response (no response).

On average, we observed that, in young neurons, about 14% of neurons show no response, 57% of them displayed a low response, and the remaining 29% of young cultures exhibited a high response. In aged neurons, 33% of them did not respond to the E protein, while 38% exhibited a low response and 29% a high response. Remarkably, the frequency of aging neurons that respond to the E protein is lower than young neurons. 

Ca^2+^ responses in neurons are complex. They are mostly mediated by Ca^2+^ release from intracellular stores and/or Ca^2+^ influx from the extracellular space that, in turn, may be mediated by either store-operated Ca^2+^ entry activated after Ca^2+^ store depletion, or the activation of alternative channels in the plasma membrane. All these processes may be largely influenced by the age of the cultures [33] and the formation of neural networks, also age-dependent [29], which are capable of spreading Ca^2+^ responses throughout synaptic activity. Accordingly, we decided to disentangle Ca^2+^ responses to the E protein by testing the influence of extracellular Ca^2+^, intracellular Ca^2+^ store content, and culture age on Ca^2+^ responses to the E protein. 

### 2.4. SARS-CoV-2 E Protein Induces Ca^2+^ Release in Young and Aged Hippocampal Neurons

**Figure 4:**
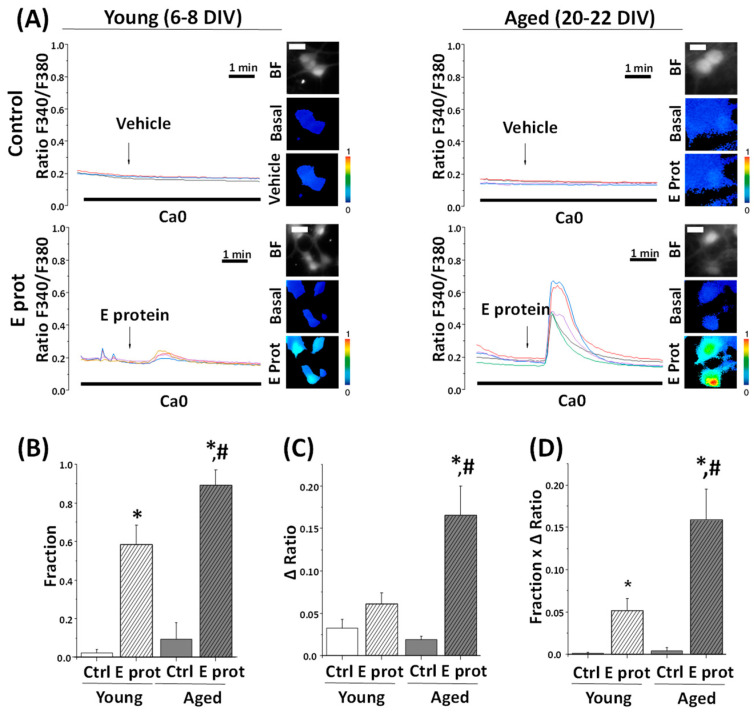
The SARS-CoV-2 E protein induces Ca^2+^ release from intracellular stores in young and aged hippocampal neurons. The release of Ca^2+^ store content was tested in hippocampal neurons from short-term (6–8 DIV) and long-term (20–22 DIV) cultures. Cells were loaded with fura2/AM (4 µM) and subjected to fluorescence Ca^2+^ imaging in external Ca^2+^-free medium. (**A**) The pictures show bright field (BF) and pseudocolor images of fluorescence ratios in basal conditions (Basal) and after vehicle (Vehicle) or E protein (E protein) treatment (2 μg/mL), in short-term (6–8 DIV) and long-term (20–22 DIV) cultured neurons. The bar represents 10 μm and applies to all photographs. Traces are representative recordings of fluorescence ratios in individual neurons identified by their morphology. (**B**) The bars correspond to the average (mean ± SEM) fraction of cells responding to vehicle or E protein, (**C**) maximum height ratio F340/F380 (Δ ratio) in responsive cells, (**D**) and the product of both parameters, n = 42, 63, 60 and 59 cells from 17 independent experiments. * *p* < 0.05 vs. control; # *p* < 0.05 vs. 6–8 DIV cultures. For (**B**–**D**) n = 42, 63, 60 and 59 cells from 17 independent experiments. * *p* < 0.05 vs. control; # *p* < 0.05 vs. young (6–8 DIV) cultures.

As our previous results suggest ER as the location of the E protein in the cell, we tested whether Ca^2+^ responses were mediated by Ca^2+^ release from the ER. To address this question, young and aged hippocampal neurons were loaded with fura2/AM and subjected to Ca^2+^ imaging in Ca^2+^-free medium. The absence of Ca^2+^ in the external environment prevents Ca^2+^ from entering from the outside by membrane channels; therefore, an increase in cytoplasmic Ca^2+^ should arise only from intracellular stores. Cells were acutely treated with E protein (2 μg/mL), or the vehicle as control. Intracellular Ca^2+^ recordings show an increase in [Ca^2+^]_cyt_ after acute presentation of the E protein in both young and aged hippocampal cultures. Quantification of the fraction of responsive cells to E protein treatment and the size of the increase in [Ca^2+^]_cyt_ in responsive cells was carried out. Interestingly, the results indicate (Figure 4) that the fraction of cells which respond to the E protein is significantly larger in aged cultures than in young ones. In addition, the size of the [Ca^2+^]_cyt_ increase in responsive cells is also significantly larger in aged neurons relative to young neurons. To quantify differences in a single parameter, we expressed the product of the fraction of responsive cells by the size of the Ca^2+^ response in responsive cells. 

This parameter accurately reflects the actual differences in the Ca^2+^ response to the E protein in aged and young neurons, this Ca^2+^ response being about three-fold larger in aged neurons than in young neurons. Accordingly, these results clearly indicate that the E protein induces Ca^2+^ release from intracellular Ca^2+^ stores in primary hippocampal neurons, an effect which is much larger in aged neurons relative to young neurons. 

### 2.5. SARS-CoV-2 E Protein Prevents Ca^2+^ Release Induced by Cyclopiazonic Acid (CPA) in Young and Aged Hippocampal Neurons

**Figure 5:**
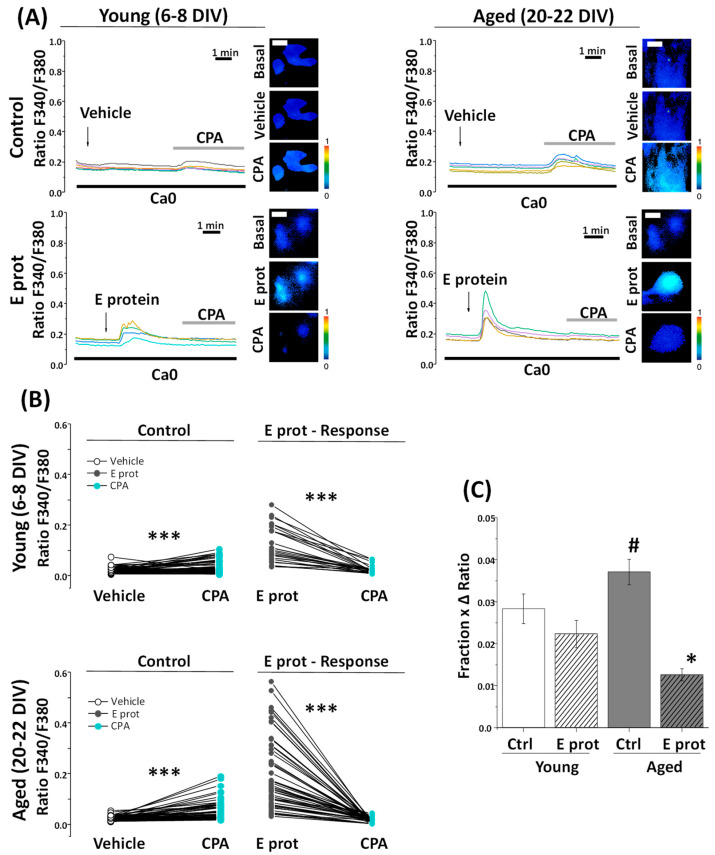
The SARS-CoV-2 E Protein prevents CPA liberation of calcium stores in aged hippocampal neurons. The release of Ca^2+^ store content was tested with CPA (10 µM) in hippocampal neurons from short-term (Young, 6–8 DIV) and long-term (Aged, 20–22 DIV) cultures. Cells were loaded with fura2/AM and subjected to fluorescence Ca^2+^ imaging in external Ca^2+^-free medium (Ca0). (**A**) The pictures show pseudocolor images of fluorescence ratios in basal conditions (Basal), after vehicle (Vehicle) or E protein (E Prot) acute treatment (2 μg/mL) and after CPA perfusion (CPA) in young and aged cultures. The bars represent 10 μm. Traces are representative recordings of fluorescence ratios in individual neurons identified by their morphology. (**B**) The line series display fluorescence paired corresponding to the CPA response after vehicle (Vehicle) or E protein (E Prot) treatment in young and aged cultures. (**C**) The bars correspond to the average (mean ± SEM) product of the fraction of cells responding and the maximum height ratio F340/F380 (Δ ratio). For (**B**,**C**) n = 42, 25, 52 and 53 from 16 independent experiments. * *p* < 0.05 vs. control; *** *p* < 0.001; # *p* < 0.05 vs. 6–8 DIV cultures.

To confirm that the E protein induces Ca^2+^ release for intracellular stores associated with the ER, we tested the effects of the SERCA pump inhibitor cyclopiazonic acid (CPA) on [Ca^2+^]_cyt_ before and after E protein presentation. Accordingly, young and aged hippocampal cultures were subjected to Ca^2+^ imaging and perfused with CPA (10 µM) after treatment with the vehicle or the E protein (2 ng/mL). Figure 5A shows representative single-cell Ca^2+^ recordings of 6–8 DIV and 20–22 DIV cultured neurons. CPA perfusion in Ca^2+^-free medium induces a [Ca^2+^]_cyt_ increase after vehicle treatment, due to the prevailing activity of ER leak channels after SERCA pump inhibition. The size of this rise is also a measure of Ca^2+^ store content. Notice that the CPA-induced [Ca^2+^]_cyt_ rise is larger in aged neurons, consistent with the larger Ca^2+^ store content reported previously by our group in aged neurons [33]. Interestingly, when CPA was added after E protein presentation, the rises in [Ca^2+^]_cyt_ induced by CPA were eliminated in both young and aged neurons. 

Pairwise data of fluorescence ratios from the same individual cells are represented in Figure 5B for young and aged cultures. Notice that both CPA and E protein-induced Ca^2+^ responses are larger in aged neurons, and E protein treatment partially decreased the CPA responses in young neurons but eliminated them in aged neurons. These results suggest that the E protein releases Ca^2+^ from intracellular stores in both young and aged neurons, but the effect appears to be stronger in aged neurons compared to young ones. 

### 2.6. Depletion of Intracellular Ca^2+^ Stores Abolishes Ca^2+^ Release Induced by the E Protein

To confirm the source of Ca^2+^ in E protein-induced rises in [Ca^2+^]_cyt_, we next tested the effects of the E protein after full depletion of intracellular Ca^2+^ stores with CPA and thapsigargin, another SERCA pump inhibitor. In contrast to CPA, Thapsigargin is an irreversible SERCA blocker. Accordingly, cells are treated with thapsigargin off the record and they keep Ca^2+^ stores empty without the need to perfuse thapsigargin throughout the experiment.

**Figure 6:**
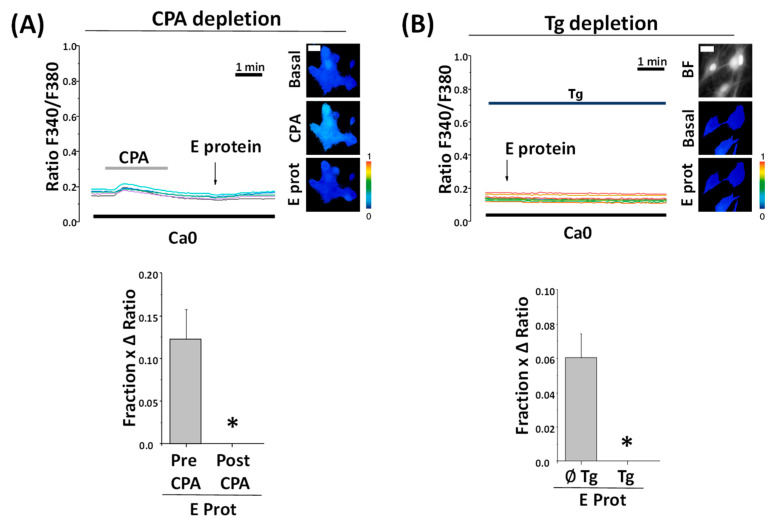
Preceding calcium stores depletion with CPA to Thapsigargin prevents SARS-CoV-2 E protein effect regardless of age. Hippocampal neurons were loaded with fura2/AM and subjected to fluorescence Ca^2+^ imaging in external Ca^2+^-free medium (Ca0). The release of Ca^2+^ store content was tested with CPA (10 µM) and thapsigargin (Tg, 1 µM). Traces are representative recordings of fluorescence ratios in individual neurons identified by their morphology. (**A**) Cells were exposed to acute treatment with the E protein (2 μg/mL) after CPA perfusion. The pictures show pseudocolor images of fluorescence ratios in basal conditions (Basal), CPA perfusion (CPA), and contiguous E protein treatment (E prot). The bars correspond to the E protein response as the product of the fraction of cells responding to treatment and the ratio of maximum height average (mean ± SEM) from cells before CPA perfusion (Pre CPA) or after (Post CPA). n = 12 and 17 cells from three independent experiments * *p* < 0.05 vs. control. (**B**) Cells were loaded with thapsigargin for 15 min and exposed to acute treatment with E protein (2 μg/mL). Pictures show bright field (BF) and pseudocolor images of fluorescence ratios in basal conditions (Basal) and after E protein treatment (E prot). The bars correspond to the E protein response as the product of the fraction of cells responding and the ratio of maximum height average (mean ± SEM) from cells previously loaded with Tg (Tg) or not (∅Tg). n = 64 and 90 from seven independent experiments. * *p* < 0.05 vs. control. The bar represents 10 μm and applies to all photographs.

In these conditions, when ER Ca^2+^ stores are depleted, the E protein failed to increase [Ca^2+^]_cyt_ at all in rat hippocampal neurons regardless of the age of the cultures, thus confirming that the E protein releases Ca^2+^ from ER-dependent, intracellular Ca^2+^ stores (Figure 6). In contrast, incubation with 2-APB, a well-known blocker of IP_3_ receptors, does not abolish Ca^2+^ responses induced by the E protein, thus suggesting that Ca^2+^ release is not mediated by the activation of protein receptors linked to phospholipase C activation but rather by the ability of the E protein to act as viroporin. However, Ca^2+^ responses to the E protein take only minutes. Accordingly, we addressed whether the dynamics of E protein translocation into neuronal endomembranes is consistent with the dynamics of the Ca^2+^ responses.

### 2.7. Dynamics of E Protein Translocation to Endomembranes

**Figure 7:**
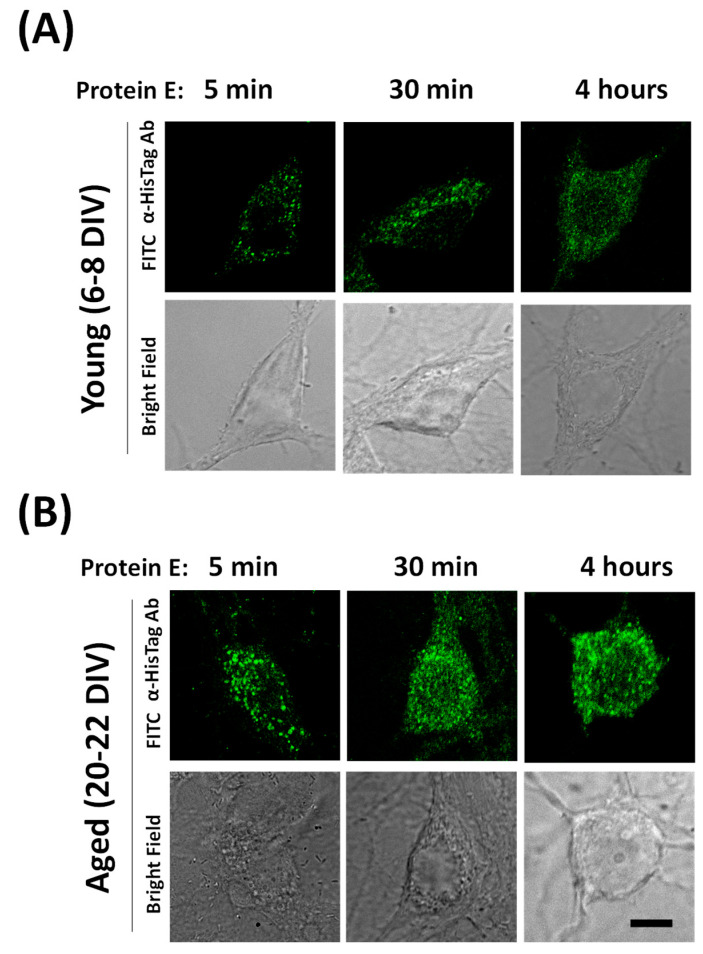
Dynamics of SARS-CoV-2 E protein translocation to endomembranes in hippocampal neurons. The dynamics of E protein location were tested in rat hippocampal cultures treated with the E protein (E protein, 2 µg/mL) for 5, 30 and 240 min (4 h) in (**A**) young (6–8 DIV) and (**B**) aged (20–22 DIV) rat hippocampal neurons. Then, cells were fixed and immunochemistry experiments were performed using E protein antibodies and confocal microscopy. Representative immunofluorescence images and the corresponding bright field images are shown. The data are representative of three similar experiments. The scale bar is 10 µm.

Our results indicate that the E protein induces Ca^2+^ release from Ca^2+^ stores at the ER in a few minutes. Accordingly, this requires the E protein to incorporate into the endomembranes of the ER in a matter of minutes. We have monitored the dynamics of E protein translocation from outside the cell to endomembranes in aged cultured neurons. For this end, we incubated the E protein with cultured neurons for 5, 30 and 240 min and then fixed the cell culture before testing the subcellular location of the E protein by means of immunofluorescence and confocal microscopy. Figure 7 shows that after only 5 min of incubation, a clear subcellular distribution of the E protein is clearly seen in the confocal image. Staining increases after 30 and 240 min. Accordingly, these data are consistent with the possibility of quickly translocating the E protein to endomembranes and releasing Ca^2+^ from the ER acting as a viroporin.

## 3. Discussion

SARS-CoV-2 caused the COVID-19 pandemic with dramatic consequences. The most important pathological effect is inflammation both at a local level, involving the lung and brain, and at a systemic level with the production of harmful cytokines. The mechanisms for neuron damage remain controversial and have not received much attention. The E protein from SARS-CoV-2 is considered a viroporin, and one of the most critical proteins for virus virulence. Viroporins are small, highly hydrophobic, virus-encoded proteins that interact with cell membranes and modify a cell’s permeability to different ions and other small molecules. The name viroporin was introduced after the discovery of different proteins from several kinds of viruses sharing common characteristics, including the binding to intracellular proteins and behavior as ion channels. It has been reported that viroporins are involved in critical steps of the viral life cycle including virion assembly and release from infected cells [13,14,34,35], as evidenced in viroporin-defective viruses that were not able to accomplish proper virus folding and release [13,14,34,36]. It has also been suggested that Viroporins are involved in virus-induced apoptosis [37]. In addition, they may also contribute to neurological damage induced by SARS-CoV-2. In fact, it is well known that SARS-CoV-2 affects mental health with neuropsychiatric complications that have been reported to be due to inflammatory mediators activated by the virus through tissue colonization of immune cells in COVID-19 [38]. For instance, olfactory dysfunction due to the SARS-CoV-2 disease can depend on the neuroinvasive properties of the virus, which could reach the olfactory bulb (OB) through the olfactory mucosa and neurons. This dysfunction, which is common in early parkinsonism, added to the evidence of cases of parkinsonism after COVID, highlight the link between SARS-CoV-2 infection and parkinsonism [39,40].

Among the 29 proteins of SARS-CoV-2, the E- and ORF3a proteins have been identified as viroporins that contribute to the massive release of inflammatory cytokines observed in COVID-19. It has previously been shown that the E proteins of both SARS-CoV and SARS-CoV-2 are able to form Ca^2+^ permeable channels, thereby triggering the activation of the NLRP3 inflammasome, leading to IL-1β overproduction [19,41]. The E proteins are well conserved within the different groups of CoVs. In SARS-CoV, the 75–76 residue containing ion channel mediates viral assembly and release. When comparing the E protein of SARS-CoV to that of SARS-CoV-2, only four amino acid exchanges at the C-terminal domain are found. Unfortunately, the effects of SARS-CoV viroporins have not been investigated in neurons despite the neurological effects of COVID-19 and SARS-CoV-2 neurotropism. 

We have investigated here whether extracellularly applied viroporin E from SARS-CoV-2 may enter cells and modify Ca^2+^ signaling and neuron cell survival. To this end, we used rat hippocampal neurons in primary culture for several reasons. In the first place, rat hippocampal neurons show a juvenile phenotype when cultured in the short term but tend to acquire an aging phenotype with a longer culture time [42]. In the second place, we have previously characterized in detail intracellular Ca^2+^ homeostasis in these neurons and the changes associated with long-term culture, resembling aging [33]. Finally, we have previously used this cell model to investigate the contribution of aging and the associated Ca^2+^ remodeling to the susceptibility to neuron damage induced by different neurotoxins, including glutamate (excitotoxicity), LPS (neuroinflammation) and amyloid-β peptide oligomers (Alzheimer’s disease) [29,31,32].

Using short-term and long-term cultures of rat hippocampal neurons, we observed that E protein treatment for 24 h leads to viroporin internalization and association with the ER. This is evidenced by confocal microscopy and the colocalization of the E protein and Tag antibodies with a specific ER tracker. Interestingly, although the E protein and tag staining colocalizes with ER, a fraction of ER does not show colocalization with the E protein. These results suggest that the SARS-CoV-2 E protein is able to enter cells and locate into specialized regions of ER endomembranes inside hippocampal neurons. This behavior is equally observed in both young and aged cultures or rat hippocampal neurons. Previous results have shown that infection with the SARS-CoV virus or transfection of viroporin E from the SARS-CoV virus in non-neuronal cells result in localization at the ER endomembranes [15,20]. However, to our knowledge, this is the first report showing that the SARS-CoV-2 E protein may enter neurons and locate at particular ER regions in primary neurons. In addition, translocation of the E protein into endomembranes takes place within a few minutes, consistent with the rapid effects of the protein on neurons.

It has been reported that viroporins are critical to viral replication and virulence. In addition, it has been suggested they may also promote cell apoptosis. Accordingly, we asked whether extracellularly applied SARS-CoV-2 viroporin E may indeed promote apoptosis in primary neurons. Our results indicate that the E protein may induce neuron apoptosis depending on the neuronal context. In fact, in short-term cultures, the E viroporin failed to promote apoptosis. However, in long-term cultures containing neurons with an aging phenotype, the E protein induces apoptosis in a large fraction of the neurons studied. These results are similar to the effects reported in the same model for other neurotoxins including NMDA, LPS and amyloid-β peptide oligomers, which promote apoptosis in aged neurons but not in young neurons [32,43]. Specifically, all three neurotoxins induce much larger Ca^2+^ responses and neuron cell death in aged rat hippocampal neurons in primary culture than in young neurons in vitro [32]. Interestingly, COVID-19 symptoms, including neurological damage, are strongly associated with aging. Therefore, our present results suggest that neurological damage associated with COVID-19 could be at least partially mediated by viroporin E and its effects on ER in neurons.

What is the mechanism, related to the ER location and enhanced by age, involved in E protein-induced neuron damage? Given the nature of the E protein as a possible viroporin permeable to Ca^2+^ ions, we decided to investigate its effects on intracellular Ca^2+^ homeostasis in both young and aged neurons. Our results show that about 70–85% of the neurons studied responded with a clear rise in [Ca^2+^]_cyt_ within 1–3 min after E protein presentation in physiological conditions. Ca^2+^ responses were highly variable, ranging either from no response, a very small and transient response, or to large and sustained Ca^2+^ responses. Although there was an observable trend towards larger Ca^2+^ responses in aged, responsive neurons, there were also more non-responsive neurons in aged neurons compared to young neurons.

To disentangle Ca^2+^ responses to E protein in neurons, we have to take into account that changes in [Ca^2+^]_cyt_ may arise from several sources. Ca^2+^ release from intracellular stores is one possible source. Ca^2+^ entry from the extracellular medium through different types of channels is another source, including pore formation, store-operated Ca^2+^ channels activated after Ca^2+^ release from intracellular stores, and all kinds of voltage- and ligand-gated Ca^2+^ channels at the neuron´s plasma membrane. Finally, neuronal cultures are able to form networks so that the activation of a single neuron in the network may recruit many connected neurons [29]. Our results indicate that the E protein primarily induces Ca^2+^ release from intracellular stores in neurons, most likely the Ca^2+^ stores at the ER. This view is supported by the evidence showing that, in the absence of extracellular Ca^2+^, the E protein still induces a clear increase in [Ca^2+^]_cyt_. In addition, after E protein presentation, SERCA inhibition with CPA, which should increase cytosolic [Ca^2+^] in neurons with filled Ca^2+^ stores, does not produce any effect. Moreover, the previous depletion of intracellular Ca^2+^ stores with either CPA or thapsigargin eliminated Ca^2+^ responses to the E protein. All together, these data indicate that the E protein induces Ca^2+^ release from intracellular stores consistently with its location at the ER endomembranes. Finally, our results also show that Ca^2+^ release induced by the E protein in primary neurons is significantly larger in aged neurons relative to young neurons. These results could be explained by two different possibilities. The first one is that the E protein enters more favorably and accumulates into aged neurons than in young neurons, leading to a larger concentration of the E protein at ER endomembranes. Our immunofluorescence studies do not support this possibility. The second possibility is that Ca^2+^ responses are larger in aged neurons because the Ca^2+^ store is larger in these cells. Consistently with this possibility, we have shown previously that the Ca^2+^ release induced by physiological agonists or the calcium ionophore ionomycin is larger in aged neurons [33]. This is also evident in the results shown here when CPA-induced Ca^2+^ release is significantly larger, again, in aged neurons compared to young ones. Therefore, the larger Ca^2+^ release induced by the E protein in aged neurons most likely reflects the larger Ca^2+^ store content in long-term cultures of rat hippocampal neurons resembling aged neurons.

We have to consider how the effects of the E protein on intracellular Ca^2+^ homeostasis may lead to neuron damage, particularly in aged neurons. The first consequence of viroporin insertion into the ER endomembranes is ER Ca^2+^ store depletion that can dramatically influence ER functional activities. A critical consequence of Ca^2+^ store depletion could be the loss of functional coupling between the ER and the mitochondria. It has been reported that a small, continuous Ca^2+^ flux from the ER to mitochondria is mandatory for the proper activation of mitochondrial dehydrogenases that keep the Krebs cycle rolling and fuel mitochondrial respiration [44]. This may be particularly true in central neurons that rely heavily on aerobic metabolism, a process that may be compromised in aged neurons [45]. As stated above, we have recently reported that aged neurons show larger Ca^2+^ stores and enhanced ER–mitochondria contacts. Interestingly, this process is also exacerbated by neurotoxins such as amyloid-β oligomers [43]. It is tempting to speculate that this process of enhanced coupling between ER and mitochondria may be a defensive mechanism intended to ensure the adequate supply of Ca^2+^ leaking from the ER into mitochondria to attend energy demands. In this scenario, E protein-dependent Ca^2+^ store depletion may shortcut this mechanism, leading to ER stress activation, autophagia, and cell death. Interestingly, the partial depletion of Ca^2+^ stores could be partially counteracted by the activation of store-operated Ca^2+^ entry, a process intended to refill Ca^2+^ stores for subsequent cell activation. However, whereas this mechanism may operate in young neurons with large store-operated Ca^2+^ entry, this Ca^2+^ entry pathway is nearly missing in aged neurons [33], thus rendering aged neurons prone to damage elicited by viroporin-induced Ca^2+^ store depletion.

We conclude that SARS-CoV-2 viroporin E may enter primary neurons and accumulate at the ER endomembrane where, acting as a Ca^2+^ permeable channel, it may induce Ca^2+^ release, thus depleting intracellular Ca^2+^ stores and promoting apoptosis, particularly in aged neurons in vitro, where enhanced Ca^2+^ store content and ER–mitochondria contacts may be required for proper oxidative metabolism. Our results may provide new insights for fighting against brain damage associated with COVID-19 and other viral infections.

## 4. Materials and Methods

### 4.1. Animals and Reagents

Wistar rat pups were obtained from the Valladolid University animal facility. Fura2/AM was from Invitrogen (Waltham, MA, USA). Fetal bovine serum was obtained from Lonza (Basel, Switzerland). Neurobasal medium, Hank’s balanced salt solution, minimal essential medium, B27, L-glutamine, and gentamicin were obtained from Gibco (Carlsbad, CA, USA). Papain solution was obtained from Worthington (Lakewood, NJ, USA). DNase I was obtained from Sigma (Madrid, Spain). Poly-D-lysine and Annexin V were obtained from BD (Franklin Lakes, NJ, USA). SARS-CoV-2 Envelope His-Avi Tag Recombinant Protein (RP-87682) and SARS Envelope Protein Polyclonal Antibody (PA1-41158) were obtained from Invitrogen (Waltham, MA, USA). HisTag monoclonal antibody (ab18184) was obtained from Abcam (Cambridge, UK). The IgG Anti-Rabbit FITC antibody (F9887) and the IgG Anti-Mouse FITC antibody (F5262) were obtained from Sigma (Madrid, Spain). Other reagents and chemicals were obtained from Sigma (Madrid, Spain) or Merck (Darmstadt, Germany).

### 4.2. Rat Hippocampal Cell Cultures

Hippocampal primary cultures were obtained from P0 Wistar rats as reported by Brewer et al. [46] with the modifications introduced by Perez-Otano et al. [47]. The rats were slaughtered by means of decapitation and the brain was removed with surgical material, transferred to filtered papain solution and incubated at 37 °C for 30 min with occasional gentle shaking. DNase I (50 µg/mL) was added to the sample 15 min after papain incubation began. Tissue pieces were mechanically dissociated into a single cell suspension. The resulting cell solution was centrifuged at 160× *g* for 4 min at 24 °C. Hippocampal cells were plated onto poly-D-lysine coated, 12 mm diameter glass coverslips at 30 × 10^3^ cells/dish. Hippocampal cultures were grown in Neurobasal Medium supplemented with B27 and 10% horse serum at 37 °C and 5% CO_2_. The duration of in vitro culture was 6–8 days in vitro (DIV) for young cultures and 20–22 DIV for aged cultures, without cell medium replacement.

### 4.3. Immunofluorescence

Hippocampal cells plated in glass coverslips were washed with 1% PBS and fixed with a solution of 4% paraformaldehyde and 4% sucrose for 15 min. After further washing with 1% PBS, cells were permeabilized with 0.1% triton x-100 for 10 min. Then, non-specific binding sites were blocked with 10% BSA for 20 min. The primary antibody for E protein or HisTag was diluted 1:500 in a PBS solution with 3% BSA and 0.1% triton x-100. Next, the cells were incubated overnight with a primary antibody solution at 4 °C in the dark. After this time and 1% PBS washing, the cultures were incubated with a secondary antibody diluted 1:1000 in a PBS solution with 3% BSA at room temperature, for 1 h in darkness. Cell nuclei were stained using Hoechst diluted 1:5000. Fluorescence images were taken using a Nikon Ni-E microscope (Nikon Instruments, Tokyo, Japan). Fluorescence measurements on individual cells were performed with the software Image J-win64 and values were expressed in arbitrary units (a.u).

### 4.4. Confocal Microscopy, Colocalization and Internalization Dynamics

Colocalization experiments were performed with a Leica TCS SP5 X confocal microscope (Leica Microsystems, Mannheim, Germany). The resulting images were analyzed with ImageJ software, and colocalization results between FITC and ER-Tracker were quantitatively measured using Manders coefficients. Confocal microscopy was also preferred for studying the dynamics of E protein translocation to endomembranes. Young and aged cultures were incubated with the E protein for 5 min, 30 min, or 4 h. Then, the immunofluorescence protocol described above was followed to fix, permeabilize, and block non-specific binding sites, followed by an overnight incubation with a primary antibody solution (E protein antibody, 1:500 in PBS with 3% BSA) at 4 °C in the dark. Next, a secondary incubation was performed with a secondary antibody solution (1:1000 in PBS with 3% BSA) at room temperature for 1 h in the dark. Fluorescence images were taken with a Leica TCS SP5 X confocal microscope (Leica Microsystems, Mannheim, Germany).

### 4.5. Apoptosis Assay

Hippocampal cells were plated in 12 mm glass coverslips at a density of 3 × 10^4^ cells/mL. On the desired DIV, the cells were incubated with E protein (0.6 µg/mL) for 24 h at 37 °C and 5% CO_2_. After treatment, cells were washed with Annexin Biding Buffer (Hepes 10 mM, NaCl 140 mM and CaCl_2_ 2.5 mM), and the cells were then incubated with Anexin V-FITC (BD Bioscience, Franklin Lakes, NJ, USA) for 15 min at room temperature and in darkness. Neuronal survival assay was performed by means of fluorescence microscopy, using an inverted microscope Nikon Eclipse TS100, and the results were given as a percentage of death. This percentage was determined for each cell culture after calculating the ratio between cells marked in green, and therefore dead, and total neurons.

### 4.6. Fluorescence Imaging of Cytosolic Ca^2+^ Concentration

Hippocampal cells cultured for 6–8 DIV or 20–22 DIV were loaded with fura-2 AM (4 µm, 1 h) in Ca1 medium containing 145 mM NaCl, 5 mM KCl, 1 mM CaCl_2_, 1 mM MgCl_2_, 10 mM glucose, and 10 mM Hepes 10 (pH 7.42). Coverslips were placed in a flow chamber and set in an inverted microscope Zeiss Axiovert S100 TV (Zeiss, Jena, Germany). The perfusion system (Warner Instruments LLC Hamden, CT, USA) works continuously and the flow temperature is maintained at 37 °C by a thermostat. For Ca^2+^ imaging, cells were illuminated by monochromatic light at 340 nm and 380 nm alternatively. To achieve this, a halogen lamp and a filter wheel (Warner Instruments LLC Hamden, CT, USA) were used. Cells were acutely exposed to either vehicle or 2 μg/mL E protein during the experiment using Ca^2+^ containing (Ca1), or free calcium medium (Ca0) as basal media, this last one containing 145 mM NaCl, 5 mM KCl, 1 mM MgCl_2_, 10 mM glucose, 10 mM Hepes and 1 mM Tris-HCl–0.5 mM EGTA 10 (pH 7.42). The cell response to N-Methyl-D-Aspartate (NMDA) medium (145 mM NaCl, 5 mM KCl, 10 mM glucose, 10 mM Hepes, 1 mM CaCl_2_, 100 µM glycine, and 100 µM NMDA). The effects of NMDA were tested at the end of the experiments to identify neurons, as glia do not respond to NMDA, and additionally to make sure cells that do not respond to the E protein are actually alive and respond to a general stimulus like NMDA. Ca^2+^ store content was measured using 10 µM cyclopiazonic acid (CPA) in Ca^2+^-free media. During the experiment, consecutive frames were captured and cytosolic Ca^2+^ concentration values from regions of interest (ROIs) corresponding to individual neurons were averaged and expressed as the ratio of fluorescence emission. The increase in cytosolic Ca^2+^ concentration was calculated by measuring the height of the calcium signal relative to baseline levels and expressed as ΔRatio (F340/F380). We consider a Δratio less than 0.06 (u.a) to mean no response, increments between 0.06 and 0.2 (u.a) a low response, and increments greater than 0.2 (u.a) a high response. The fraction of responsive cells was calculated by dividing responsive cells according to the above criteria for the total cell number in the field, considering responsive cells the ones showing a change in the slope of the Ca^2+^ recording after application of the stimulus. The fraction × ΔRatio parameter, as a measure of calcium response, was obtained by calculating the product of the previous parameters.

### 4.7. Statistical Analysis

Changes in the fluorescence ratio are expressed as ΔRatio (Ratio F340/F380). Calculation of the ΔRatio was performed using Origin 2019. Data are presented as mean ± SEM. When comparing two groups, Student’s *t*-test was used either for paired samples or independent samples, as appropriate. A two-way ANOVA with Tukey’s post hoc test was used to compare more than two groups. Differences were considered significant at *p* < 0.05.

## Data Availability

All data regarding results are available upon request to the corresponding author.
